# Aripiprazole Lauroxil Every 2 Months for the Treatment of Adults With Schizophrenia: A Post Hoc Analysis of Efficacy by Baseline Severity of Illness From Phase 3b Clinical Trial Data

**DOI:** 10.1093/schizbullopen/sgaf023

**Published:** 2025-10-15

**Authors:** John M Kane, James A McGrory

**Affiliations:** The Donald and Barbara Zucker School of Medicine at Hofstra/Northwell, Hempstead, NY 11549, United States; Alkermes, Inc., Waltham, MA 02451-1420, United States

**Keywords:** activation factor, efficacy, second-generation antipsychotic

## Abstract

**Background:**

This post hoc analysis examined the efficacy of aripiprazole lauroxil (AL) by baseline severity of illness in the double-blind Aripiprazole Lauroxil and Paliperidone palmitate: INitiation Effectiveness study (NCT03345979) in patients with schizophrenia treated with AL every 2 months.

**Study Methods:**

Adults with acute schizophrenia were randomized to AL 1064 mg every 2 months or active control (paliperidone palmitate [PP] 156 mg monthly). Based on Clinical Global Impression–Severity scores, baseline severity of illness was categorized as moderate, marked, or severe. Changes from baseline in Positive and Negative Syndrome Scale (PANSS) Total score were assessed at week 25, along with PANSS items related to hostility/excitement. Numbers of patients with activation adverse events (AEs; anxiety, agitation, and insomnia) were also evaluated.

**Study Results:**

Of 99 patients assigned to AL, 31 (31%) were moderately ill at baseline, 54 (55%) were markedly ill, and 14 (14%) were severely ill. With AL treatment, mean ± SE changes from baseline in PANSS Total score at week 25 were −21.1 ± 2.5 (moderately ill; baseline, 87.1), −24.1 ± 1.8 (markedly ill; baseline, 95.3), and −25.6 ± 6.4 (severely ill, baseline, 106.1). Improvements from baseline in PANSS scores related to hostility/excitement items were comparable among severity subgroups. No clear pattern of occurrence of the AEs anxiety, agitation, and insomnia was observed across baseline severity groups.

**Conclusions:**

In this post hoc analysis, safety related to activation and efficacy with AL treatment were comparable across baseline severity-of-illness subgroups of patients with schizophrenia.

## Introduction

Schizophrenia is a severe mental illness often characterized by acute exacerbations and relapse.^1^ Treatment with long-acting injectable (LAI) antipsychotics, which provide consistent long-term medication coverage,^2^ is associated with a reduced risk of relapse and rehospitalization.^3^^,^^4^ Aripiprazole lauroxil (AL [Aristada®, Alkermes, Inc., Waltham, MA, USA]) is an atypical antipsychotic LAI approved by the US Food and Drug Administration for the treatment of schizophrenia in adults. The efficacy of AL administered every 2 months was assessed in patients hospitalized with an acute exacerbation of schizophrenia and followed through discharge to outpatient care in the randomized, controlled, phase 3b ALPINE (Aripiprazole Lauroxil and Paliperidone palmitate: INitiation Effectiveness) study.^5^ Significant within-group improvements from baseline in Positive and Negative Syndrome Scale^6^ (PANSS) Total score at weeks 4, 9, and 25 were achieved in patients treated with AL or paliperidone palmitate^5^ (PP; included as an active control with known effectiveness^7^).

Real-world data indicate that LAIs are offered more commonly to patients with severe illness, but patients may benefit from their use regardless of their baseline severity.^8^ In addition to improving medication adherence and reducing discontinuation rates,^9–11^ treatment with LAI antipsychotics is associated with improved long-term outcomes.^3^^,^^4^^,^^10–13^ This post hoc analysis of data from ALPINE examined the efficacy of the LAI AL in subgroups of patients with schizophrenia who were moderately, markedly, or severely ill at baseline. Because symptoms related to hostility, excitement, or activation are associated with poor treatment adherence^14^^,^^15^ and an increased risk for inpatient admissions,^16^ the PANSS activation score was also assessed.

## Methods

Data from the randomized, double-blind, active-controlled, phase 3b ALPINE study (ClinicalTrials.gov identifier NCT03345979) were used in this post hoc analysis. Study sites included 16 US clinical research centers. ALPINE study methods, summarized here, have been reported previously.^5^ The study protocol was approved by site-specific independent ethics committees/institutional review boards and was conducted in accordance with the principles of Good Clinical Practice derived from the Declaration of Helsinki and in accordance with local regulations and the International Conference on Harmonisation guidelines. All patients (or their legal representative) provided written informed consent before any study-specific procedures were conducted.

### Patients and Study Design

**Figure 1:**
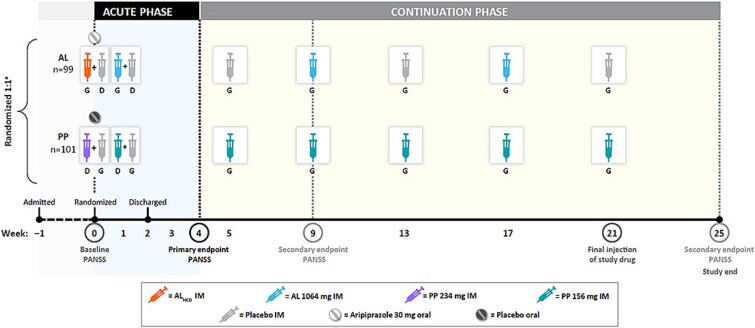
ALPINE Study Design. ^a^Because AL initiation required gluteal injection and PP initiation required deltoid injection, placebo injections were administered in patients’ deltoid and gluteal muscles, respectively, during initiation to maintain blinding. The AL group also received a placebo injection at weeks 5, 13, and 21 to match the PP dosing schedule, and the PP group received an oral placebo tablet on day 1 to match the oral dose of aripiprazole in the AL initiation regimen. Adapted with permission from Weiden et al.^5^ Abbreviations: AL = aripiprazole lauroxil; AL_NCD_ = NanoCrystal dispersion formulation of AL; ALPINE = Aripiprazole Lauroxil and Paliperidone palmitate: INitiation Effectiveness; D = deltoid; G = gluteal; IM = intramuscular; PANSS = Positive and Negative Syndrome Scale; PP = paliperidone palmitate

Adult patients (aged 18-65 years) with an acute exacerbation of schizophrenia (according to the *Diagnostic and Statistical Manual of Mental Disorders, Fifth Edition*^17^) were enrolled as inpatients, randomized to treatment, discharged after 2 weeks, and then followed as outpatients through week 25 (Figure 1). Eligible patients had a PANSS Total score of 80-120 (inclusive) and Clinical Global Impression–Severity^18^ (CGI-S) score of at least 4 at screening and at baseline. Eligible patients also scored 4 or higher on 2 or more of the following PANSS Positive scale items: delusions, conceptual disorganization, hallucinatory behavior, or suspiciousness/persecution. Patients with a history of hypersensitivity or intolerance to aripiprazole, risperidone, or paliperidone were excluded; patients without previous exposure received test doses during screening to assess tolerability.

Patients were assigned to receive AL 1064 mg every 2 months or the active control, PP 156 mg monthly. AL was initiated using a 1-day regimen including a single intramuscular (IM) 675-mg injection of a NanoCrystal Dispersion formulation of AL (AL_NCD_; Aristada Initio [Alkermes, Inc]) and a single 30-mg oral dose of ari-piprazole on day 1. An IM injection of AL 1064 mg was then administered on day 8 and every 8 weeks thereafter. Patients assigned to PP received a 234-mg IM injection and an oral placebo tablet on day 1 and a 156-mg IM injection on day 8 and every 4 weeks thereafter. Placebo injections were used to maintain study blinding; timing of the injections is shown in Figure 1.

The PANSS and CGI-S were administered at baseline, day 4, weeks 1 and 2, and at regularly scheduled outpatient visits. Primary and secondary efficacy endpoints in ALPINE were changes from baseline in PANSS Total score at week 4 and at weeks 9 and 25, respectively.^5^ Adverse events (AEs) were assessed throughout the study.

### Post Hoc Outcomes and Analysis

To characterize effects of AL treatment on symptoms of schizophrenia (mean change in PANSS scores) in patients who were moderately, markedly, or severely ill at baseline, severity of illness was categorized according to baseline CGI-S score (CGI-S = 4, CGI-S = 5, or CGI-S = 6, respectively).^18^ The activation/excitement symptom dimension was assessed using a 5-item activation factor composed of the excitement and hostility items from the PANSS Positive subscale and anxiety, tension, and poor impulse control items from the PANSS General Psychopathology subscale.^19^ The possible scoring range for the activation factor was 5-35. Changes from baseline in PANSS Total and activation scores at day 4 and weeks 1, 2, 4, 9, and 25 were assessed by baseline severity.

A hypothetical concern has been raised in the literature that onset of treatment with aripiprazole could be associated with increased agitation or activation in some patients with schizophrenia.^20–22^ Therefore, an assessment of activation AEs was included in this analysis. To determine whether their occurrence might differ with baseline severity of illness, the numbers of patients in each baseline severity subgroup who reported AEs related to activation symptoms were also evaluated.

The post hoc analysis included all randomized patients who received at least 1 dose of study drug; efficacy was analyzed for patients who had at least 1 postbaseline PANSS assessment. Mean (SE) changes from baseline at each time point were calculated for PANSS Total and activation factor scores by baseline severity group. AL and PP were analyzed separately because the study was not powered for formal statistical comparisons between groups; the PP arm provided an active control. No statistical testing was conducted in this analysis; only summary statistics were calculated.

## Results

A total of 200 patients received at least 1 dose of AL (*n* = 99) or PP (*n* = 101) in ALPINE. Of patients assigned to AL, 31 (31%) were moderately ill at baseline, 54 (55%) were markedly ill, and 14 (14%) were severely ill (PP, 26/101 [26%], 59/101 [58%], and 16/101 [16%], respectively). Patients assigned AL treatment had a mean (SD) age of 43.5 (9.7) years and body mass index of 28.2 (5.5) kg/m^2^ at baseline (PP, 43.4 [10.8] years and 27.9 [5.1] kg/m^2^, respectively). Table 1 lists patient demographics and baseline clinical characteristics by baseline severity of illness.

**Table 1:** Demographics and Baseline Clinical Characteristic Scores by Baseline Severity Group^a^

Characteristics	Moderately ill	Markedly ill	Severely ill
Aripiprazole lauroxil, n	31	54	14
Age, mean (SD), years	46.4 (9.9)	42.5 (8.9)	41.1 (11.2)
BMI, mean (SD), kg/m^2^	29.0 (4.8)	28.5 (5.9)	25.3 (5.0)
PANSS Total score,^b^ mean (SD)	87.1 (5.5)	95.3 (7.3)	106.1 (7.2)
Activation factor score,^b^ mean (SD)	12.3 (3.0)	14.4 (3.4)	16.3 (3.0)
Paliperidone palmitate, *n*	26	59	16
Age, mean (SD), years	44.3 (12.8)	42.4 (10.1)	45.3 (10.4)
BMI, mean (SD), kg/m^2^	28.4 (5.2)	28.1 (5.1)	25.9 (4.8)
PANSS Total score,^c^ mean (SD)	88.7 (5.1)	94.6 (7.6)	104.6 (6.1)
Activation factor score,^c^ mean (SD)	12.9 (3.0)	14.4 (3.5)	16.0 (2.1)

Abbreviations: AL = aripiprazole lauroxil; BMI = body mass index; CGI-S = Clinical Global Impression–Severity; PANSS = Positive and Negative Syndrome Scale; PP = paliperidone palmitate.

a Moderately ill, CGI-S = 4; markedly ill, CGI-S = 5; severely ill, CGI-S = 6.

b Efficacy analysis: AL, moderately ill, *n* = 31; markedly ill, *n* = 52; severely ill, *n* = 13.

c Efficacy analysis: PP, moderately ill, *n* = 26; markedly ill, *n* = 57; severely ill, *n* = 16.

**Figure 2:**
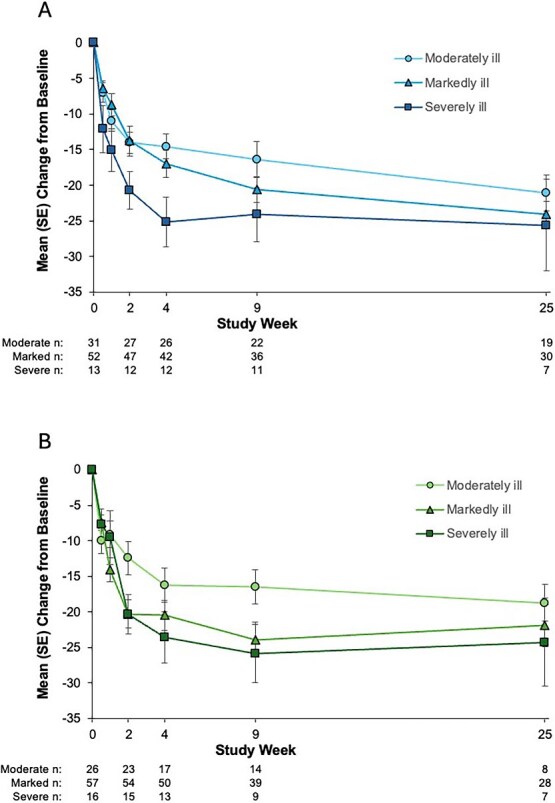
Changes From Baseline in PANSS Total Score by Baseline Severity Group.^a^ (A) Aripiprazole lauroxil; (B) paliperidone palmitate. ^a^Moderately ill, CGI-S = 4; markedly ill, CGI-S = 5; severely ill, CGI-S = 6. Abbreviations: CGI-S = Clinical Global Impression–Severity; PANSS = Positive and Negative Syndrome Scale

PANSS Total scores improved during treatment with AL or PP regardless of patients’ baseline severity of illness (Figure 2). After 4 weeks of AL treatment, mean (SE) changes in PANSS Total score in the moderately, markedly, and severely ill subgroups were −14.5 (1.7), −17.0 (1.9), and −25.2 (3.5), respectively, from baseline mean (SD) scores of 87.1 (5.5), 95.3 (7.3), and 106.1 (7.2). After 25-week AL treatment, mean (SE) changes in PANSS Total score were −21.1 (2.5), −24.1 (1.8), and −25.6 (6.4) in the moderately, markedly, and severely ill subgroups, respectively. Mean (SE) changes in PANSS Total score observed during PP treatment were −16.2 (2.4), −20.5 (2.1), and −23.5 (3.6) at week 4 and −18.8 (2.6), −21.9 (2.9), and −24.3 (6.2) at week 25 in the moderately, markedly, and severely ill subgroups, respectively.

**Figure 3:**
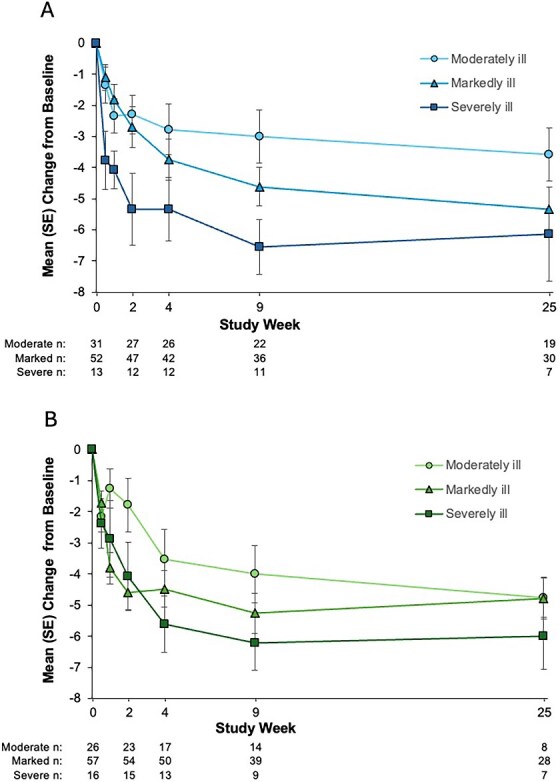
Changes From Baseline in PANSS Activation Factor Score by Baseline Severity Group.^a^ (A) Aripiprazole lauroxil; (B) paliperidone palmitate. ^a^Moderately ill, CGI-S = 4; markedly ill, CGI-S = 5; severely ill, CGI-S = 6. Abbreviations: CGI-S = Clinical Global Impression–Severity; PANSS = Positive and Negative Syndrome Scale

Mean (SD) activation factor scores were relatively low at baseline, ranging from 12.3 to 16.3 (possible scoring range, 5-35)^6^ across AL and PP illness severity groups (Table 1). Decreases in activation factor scores were nonetheless observed in all severity subgroups (Figure 3). Among moderately, markedly, and severely ill patients treated with AL, mean (SE) changes in activation factor score at week 4 were −2.8 (0.8), −3.7 (0.7), and −5.3 (1.0), respectively. Mean (SE) changes in activation score at week 25 were −3.6 (0.9), −5.3 (0.7), and −6.1 (1.5), respectively. For PP, mean (SE) changes in activation factor score were −3.5 (1.0), −4.5 (0.6), and −5.6 (0.9) at week 4 and −4.8 (0.6), −4.8 (0.7), and −6.0 (1.1) at week 25 among moderately, markedly, and severely ill patients, respectively.

AEs related to activation included anxiety, agitation, and insomnia. Seven AL-treated patients and 10 PP-treated patients reported 1 or more activation AE (Table 2). No pattern of events related to baseline severity of illness was observed during treatment with either AL (moderate, 3/31 [10%]; marked, 3/54 [6%]; severe 1/14 [7%]) or PP (moderate, 5/26 [19%]; marked, 2/59 [3%]; severe, 3/16 [19%]).

**Table 2:** AEs Related to Activation Symptoms by Baseline Severity Group^a^

AEs, n (%)	Moderately ill	Markedly ill	Severely ill
Aripiprazole lauroxil, n	31	54	14
Anxiety	2 (6.5)	0	0
Agitation	0	2 (3.7)	1 (7.1)
Insomnia	1 (3.2)	1 (1.9)	0
Paliperidone palmitate, n	26	59	16
Anxiety	4 (15.4)	0	3 (18.8)
Agitation	0	1 (1.7)	0
Insomnia	1 (3.8)	1 (1.7)	1 (6.3)

Abbreviations: AE = adverse event; CGI-S = Clinical Global Impression–Severity.

a Moderately ill CGI-S = 4; markedly ill, CGI-S = 5; severely ill, CGI-S = 6.

## Discussion

In this post hoc analysis of data from the ALPINE study, acutely ill patients with schizophrenia who initiated AL 1064 mg every 2 months using a 1-day initiation regimen and were transitioned to outpatient care experienced improvement in schizophrenia symptoms regardless of their baseline severity of illness. A similar pattern of change in PANSS Total scores across severity subgroups was observed with the PP 156-mg monthly active control. Decreases in mean activation factor scores with AL or PP treatment also were observed in all baseline severity subgroups.

Change from baseline in PANSS Total score was numerically greater for the most severely ill subgroup, consistent with findings from a meta-analysis of patient-level data from placebo-controlled trials.^23^ In that analysis, treatment benefits with medium-to-large effect sizes were observed for moderately to severely ill patients with schizophrenia (based on PANSS scores anchored to CGI-S ratings of 4, 5, or 6), and both change from baseline in PANSS Total score and treatment effect size increased with baseline severity of illness.^23^ In the current analysis, differences in illness severity between subgroups were reduced but not eliminated after 25-week treatment with AL or PP. For AL, the 4.5-point greater mean improvement in PANSS Total score for severely ill versus moderately ill patients at week 25 did not overcome the 19.0-point disparity between group means at baseline. PP-treated patients had a mean difference of 15.9 points between severely and moderately ill groups at baseline and a mean 5.6-point difference in improvement at week 25. The baseline difference in PANSS activation factor score improvements between the moderately and severely ill groups also was not eliminated by the greater improvement in the severely ill group. Change from baseline in PANSS Total score for each subgroup was within approximately 2 points of the overall mean for AL (−23.3) and within approximately 3 points over the overall mean for PP (−21.7) by week 25.^5^

The transition to outpatient care after hospitalization for an acute exacerbation of schizophrenia can be challenging. For patients with less severe symptoms, a short duration of inpatient stay may be a barrier to initiation of an LAI medication, which may not be fully initiated at the time of discharge if extended oral supplementation is required.^24^ Gaps in medication after discharge can increase the risk of rehospitalization,^25^ and more severe symptoms of schizophrenia, particularly activation/excitement symptoms, are associated with poor adherence.^14^^,^^15^ Although treatment with LAI antipsychotics may reduce patients’ risk of relapse and rehospitalization,^3^^,^^4^ the proportion of patients with schizophrenia who receive an LAI medication remains low—in a real-world study of mostly commercially insured patients in the United States, only 4% of patients newly diagnosed with schizophrenia were treated with an LAI.^26^ Safety and efficacy findings from the ALPINE study suggest that AL every 2 months is an appropriate treatment option for inpatients with schizophrenia, and starting AL with a 1-day regimen of a single AL_NCD_ injection and a 30-mg oral dose of aripiprazole enables LAI initiation during even a short inpatient stay.^5^ Results from this post hoc analysis further indicate that AL can effectively reduce symptoms of schizophrenia among patients at either end of the severity spectrum, including both moderately ill patients requiring just a short inpatient stay and severely ill patients, who may be at risk for nonadherence to oral medication.

PANSS scores are widely used to comprehensively assess changes in schizophrenia psychopathology during treatment in clinical trials but are measured on a continuous scale.^27^ In this analysis, the categorical CGI-S was used to assign baseline severity subgroups because it allows grouping of patients according to a clinical judgement of their overall severity of illness^28^; this grouping enabled characterization of symptom reduction within each subgroup. There was overlap in baseline PANSS Total scores across severity groups, particularly among patients in the moderately and markedly ill categories; however, the mean baseline PANSS Total score was higher in each successive severity category. A limitation of this analysis was that treatment assignment was not stratified by baseline severity of illness and the numbers of patients in some of the severity-of-illness subgroups were small, which may limit interpretability of the results. Although outside the scope of the present analysis, an assessment of how degrees of symptom improvement varied with baseline severity as a continuous measure would be of interest for future research.

In this post hoc analysis of ALPINE data, AL treatment reduced symptoms of schizophrenia among patients in each baseline severity subgroup. For both the AL and the active control PP arms, changes from baseline in PANSS Total score at week 25 by baseline severity group were comparable with those observed in the overall study. Improvement from baseline in activation factor score was also observed among patients with moderate, marked, or severe symptoms at baseline. Few AEs of anxiety, agitation, or insomnia were reported during AL or PP treatment, and no clear pattern of occurrence of those AEs was observed across baseline severity groups. These results suggest that AL is a safe and effective treatment option for patients with acute symptoms of schizophrenia, regardless of baseline severity of illness, who are initiated on LAI antipsychotic treatment as inpatients and transitioned to outpatient care, including severely ill patients with schizophrenia and moderately or markedly ill patients who would be likely to receive an oral antipsychotic but might have improved long-term outcomes with LAI treatment.
